# The Level of Antibodies to Tumor-Associated Glycans in Gastric Cancer Patients Is Lower than in Healthy Donors and Reduces with Age

**DOI:** 10.3390/ijms27020800

**Published:** 2026-01-13

**Authors:** Maxim P. Nikulin, Alexander D. Lipatnikov, Alexei Yu. Nokel, Svetlana M. Polyakova, Svetlana V. Tsygankova, Galina V. Pazynina, Alexandra V. Semyanikhina, Elena V. Ogorodnikova, Dmitry V. Rogozhin, Olesya M. Rossomakhina, Dmitrii A. Atiakshin, Olga I. Patsap, Ivan S. Stilidi, Nicolai V. Bovin, Igor Buchwalow, Markus Tiemann, Nadezhda V. Shilova

**Affiliations:** 1 Federal State Budgetary Institution “National Medical Research Center of Oncology Named After N.N. Blokhin” of the Ministry of Health of the Russian Federation, 23, Kashirskoe Shosse, Moscow 115522, Russia; alexandra_silina@mail.ru (A.V.S.); ogorodnikovaelena@yandex.ru (E.V.O.); d.rogozhin@ronc.ru (D.V.R.); i.stilidi@front.ru (I.S.S.); 2 Shemyakin-Ovchinnikov Institute of Bioorganic Chemistry of the Russian Academy of Sciences, 16/10, Miklukho-Maklaya Street, Moscow 117997, Russia; alex.9508@yandex.ru (A.D.L.); smpolyak@googlemail.com (S.M.P.); svlush@mail.ru (S.V.T.); pazininagv@gmail.com (G.V.P.); professorbovin@yandex.ru (N.V.B.); pumatnv@gmail.com (N.V.S.); 3 Federal State Budgetary Institution “National Medical Research Center for Obstetrics, Gynecology and Perinatology Named After Academician V.I. Kulakov” of the Ministry of Health of the Russian Federation, 4, Oparina Street, Moscow 117997, Russia; anokel@internet.ru; 4 Federal State Budgetary Scientific Institution “Research Centre of Medical Genetics”, 1, Moskvorechye Street, Moscow 115522, Russia; 5 Federal State Budgetary Educational Institution of Higher Education “Lugansk State Medical University Named After St. Luke” of the Ministry of Health of the Russian Federation, 1G, Quarter of the 50th Anniversary of the Defense of Lugansk, Lugansk 291045, Russia; lesya_ros@mail.ru; 6 Research and Educational Resource Center for Immunophenotyping, Digital Spatial Profiling and Ultrastructural Analysis Innovative Technologies, Peoples’ Friendship University of Russia, 6, Miklukho-Maklaya Str., Moscow 117198, Russia; atyakshin-da@rudn.ru (D.A.A.); olga@patsap.ru (O.I.P.); buchwalow@pathologie-hh.de (I.B.); 7 Institute for Hematopathology, Fangdieckstr, 75a, 22547 Hamburg, Germany; mtiemann@hp-hamburg.de

**Keywords:** gastric cancer, anti-glycan antibodies, IgM, printed glycan array, tumor-associated carbohydrate antigens

## Abstract

A key function of naturally occurring antibodies is to control pathologically altered cells, such as those with aberrant glycosylation. Age-related diminution in the pool of B cells producing these immunoglobulins is linked to impaired anti-tumor immunity. In this study, the levels of antibodies against tumor-associated carbohydrate antigens (TACAs)—common in gastric cancer (GC) and other malignancies—were analyzed in 235 treatment-naïve GC patients (stages I–IV) and 76 healthy donors using a printed glycan array (PGA). We found that anti-glycan IgM levels, but not IgG, reduced with age in both patients and donors. Crucially, IgM levels against most glycans were significantly lower in the GC cohort compared with healthy donors, a trend that remained after age adjustment. Furthermore, an immunohistochemical analysis revealed that human anti-GalNAcα (Tn) antibodies—a well-characterized TACA in gastrointestinal cancers—bound to tumor cells and exhibited perinuclear and membrane staining in non-tumor surface cells within the same organ. These data support the hypothesis that gastric cancer patients have reduced levels of anti-glycan IgMs, which are responsible for the early recognition of transformed cells. This specific immunodeficiency may contribute to a permissive environment for tumor development.

## 1. Introduction

One of the key components of anti-tumor immunity is naturally occurring antibodies (nAbs)—immunoglobulins that emerge in the body during the first years of life as a result of B1-cell stimulation by gastrointestinal (GI) microbiota [1]. These antibodies belong mainly to the IgM class. One of their main functions is the clearance of pathologically altered cells; the specificity and efficiency of this process support the continuing interest in anti-tumor nAbs [2,3], some of which target glycans.

Glycans play a crucial role in various physiological processes: they participate, for instance, in cell signaling, protein folding and leukocyte–endothelial adhesion. Heavily glycosylated mucins line the mucous membranes of the GI tract, bronchi, and urogenital tract, protecting cells from harmful agents while serving as a nutrient substrate and habitat for microbiota [1]. During tumor transformation, cellular glycans undergo shortening or elongation, hyper- or hypo-sialylation, and sometimes the synthesis of neo-antigens, which are found in healthy cells only in vanishing quantities or are characteristic of completely different organs. Such aberrant glycans have been termed tumor-associated carbohydrate antigens (TACAs) [4,5]. Among the most well-known TACAs are the GalNAcα (Tn) and Galβ1-3GalNAcα (TF) antigens, which appear on GI mucins. Both of these glycans are involved in cell adhesion and detected in gastric, pancreatic, colorectal, lung, and other cancers [2,6,7,8,9,10]. The glycoantigen sialyl-Le^A^ is known as CA19-9, while sialyl-Tn is part of CA72-4; both are widely used markers for diagnosing and monitoring digestive tract tumors [11]. To date, dozens of TACAs have been identified [12].

Several studies have attempted to compare anti-TACA antibody levels between cancer patients and healthy donors. It has been shown that low levels of anti-Tn and anti-TF antibodies correlate with advanced disease stages, poor treatment response, unfavorable prognosis, and aging [8,13,14]. Another study demonstrated reduced levels of IgG and IgM antibodies against TF and MUC1 antigens in gastric cancer (GC) patients compared with healthy donors, though notably, the median age of the patients (65 years) differed significantly from that of the donors (51 years) [15]. Other researchers analyzed the anti-glycan antibody (AGA) level in colorectal cancer patients and healthy donors, where patient ages ranged from 43 to 88 years (median not specified) but the donor age distribution was not detailed [16].

Advances in glycan array technology (printed and suspension glycan arrays) and cohort studies have led to the identification of hundreds of AGAs in human blood, some of which are classified as anti-TACA antibodies [17,18,19,20,21]. Another study thoroughly examined the influence of age, sex, race, and blood type on IgG and IgM AGAs detected via glycan arrays [22]. It was found that IgM levels in the 20–24 and 25–29 age groups significantly differed from those in the 40–44 and 50–65 age groups, whereas no such difference was observed for IgG. Interestingly, women tended to have higher AGA-IgM than men, though the difference was not significant [22]. An earlier study on antibodies targeting the trisaccharide Galα1-3Galβ1-4GlcNAcβ (the Galili xenoantigen) in healthy donors reported significant age-related difference for IgM, but not IgG [23].

Thus, the existing literature indicates that AGA levels—particularly IgM—decline with age in healthy donors. However, it remains unclear whether similar age-related changes occur in cancer patients and whether these changes are a natural process or influenced by the tumor itself.

GC ranks among the leading causes of morbidity and mortality worldwide. In 2022, 968,350 new GC cases were diagnosed globally, with 659,853 deaths [24]. A significant proportion of patients are still only diagnosed at advanced stages [25]. The primary treatments for resectable GC (about 60% of diagnosed cases) are surgery and combined therapy, yet even after full treatment, patients cannot be considered as being fully cured. Immune checkpoint inhibitor therapy is used in GC only in limited cases [26]. However, SC-1, a monoclonal IgM antibody to glycans obtained from B1 cells of patients with GC, is close to clinical use. SC-1 has been shown to induce tumor cell death via lipoptosis [27], and studies are underway to explore its therapeutic potential in GC patients [28,29,30].

Considering the above, studying the relationship between the pathogenesis of GC and nAbs is promising for advancing early diagnosis and treatment. In this study, we evaluated AGA levels against known and potential TACAs in a cohort of GC patients and conditionally healthy donors using a printed glycan array (PGA) and analyzed their correlation with age.

## 2. Results

### 2.1. PGA Printing and Quality Control

To obtain reliable results, the PGA underwent a quality control procedure whose main stages were developed in previous work [31]. The first stage was performed using the sciFLEXARRAYER robot’s quality control system to exclude missing ligand spots. The second stage involved visual inspection of the obtained images and verification of immobilized glycan functionality and result reproducibility by developing randomly selected PGAs (at least 4% from each batch) with standard human plasma (i.e., plasma from a conditionally healthy donor; identical for all control arrays). Reproducibility was assessed by calculating the intra- and inter-slide correlation coefficients (CCCs and OCCCs, respectively) of antibody signals bound to glycans, as described in [31]. Arrays with correlation coefficients below 0.8 or showing critical imaging defects were excluded from consideration, while the remaining arrays were used for analysis of antibody levels in GC patients and healthy donors.

Analysis of AGA level using PGA: The AGA level (expressed as RFU) was measured in blood serum from 235 patients and 76 conditionally healthy donors (complete data provided in Supplementary S1). For analysis, from approximately 300 glycans of the array, we selected the well-known TACAs and some related glycans, as well as AGAs which, judging by our preliminary data and literature reports, seem promising as cancer markers. Only AGAs showing significant signals (see Materials and Methods) in at least one subgroup were taken into account. Table 1 and Table 2 present the structures and characteristics of the glycans (selected TACAs) included in the PGAs used.

In this study, we only considered IgG and IgM antibodies to TACAs whose signals exceeded threshold values across the entire cohort (see Materials and Methods). Ultimately, the analysis included 23 glycans for IgM and 8 glycans for IgG (Supplementary S2).

### 2.2. IgM

The data on median RFU values, reflecting IgM levels in GC patients and healthy donors, as detected by PGA, are presented in Supplementary S2, Table S1. Immunoglobulin M levels against all analyzed glycans were lower in patients than in healthy donors, with nearly all differences being statistically significant (*p* < 0.05).

**Figure 1:**
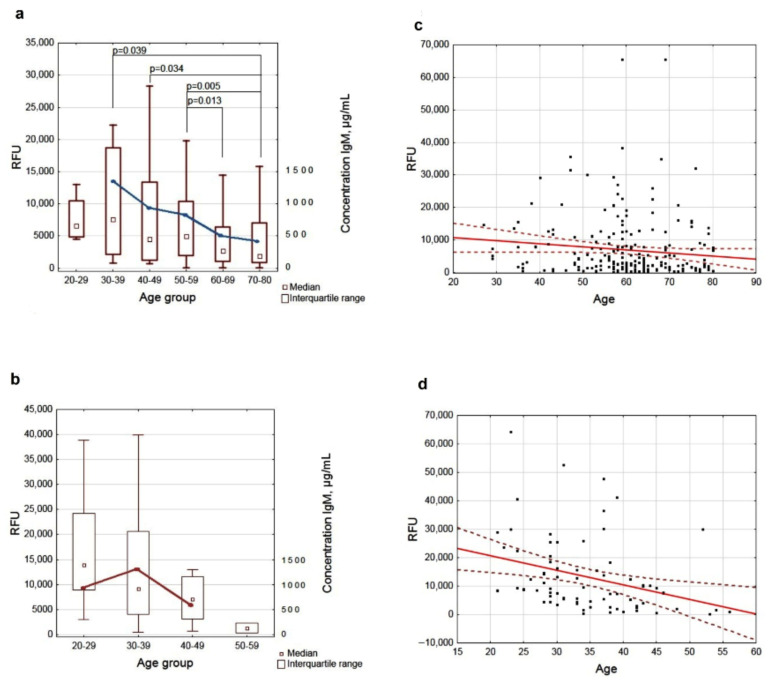
(**a**) Age-related changes in IgM to Galβ1-3GlcNAcβ (Le^C^) and total IgM level in patients with GC. The blue line demonstrates the reduction in total IgM concentration in pooled serum samples from each age subgroup. (**b**) Age-related changes in IgM to Galβ1-3GlcNAcβ (Le^C^) and total IgM level in healthy donors. The red line demonstrates the reduction in total IgM concentration in pooled serum samples from each age subgroup. The *p*-value indicates statistical significance when comparing AGA level between different age subgroups. (**c**) Regression plots with confidence intervals showing the age-dependence of the anti-Le^C^ IgM level in GC patients (ρ = −0.198, *p* = 0.002) and healthy donors (ρ = −0.419, *p* < 0.001). (**d**) Regression plots with confidence intervals showing the age-dependence of anti-Le^C^ IgM level in GC patients (ρ = −0.198, *p* = 0.002) and healthy donors (ρ = −0.419, *p* < 0.001).

For comparative analysis of anti-glycan IgM by age, both patients and donors were stratified into 10-year age subgroups, with median signal values and interquartile ranges calculated for each (Supplementary S2, Tables S2 and S3). The highest AGA level against all studied glycans was observed in the youngest age group (20–29 years) for both patients and healthy donors, followed by a progressive age-related decline. Statistically significant differences in GC groups were only observed for IgM against glycan Nos. 41, 78, 85, 88, 113, 254, 299, and 267 (Supplementary S2, Table S2; glycan structures are shown in Table 1 and Table 2). Representative examples in Figure 1a,b illustrate age-dependent changes in antibody level against TACA No. 85 (Galβ1-3GlcNAcβ, Le^C^).

In addition to evaluating the AGA level (by signal intensity), we quantified the total IgM concentration in the age subgroups (excluding small sample groups) by pooling serum samples from 11 randomly selected individuals per subgroup. The age-related changes in antibody concentration for patients and healthy donors are shown in Figure 1a,b; these demonstrate that both specific anti-Le^C^ antibodies and total IgM level significantly reduced with age in both groups. Notably, the total IgM concentration in donors aged 30–39 years was slightly higher than in those aged 20–29 and 40–49, likely reflecting natural variability in the blood IgM level.

A correlation analysis revealed weak negative associations between AGA-IgM level and age in GC patients (ρ = −0.20 to −0.10). These correlations were statistically significant for half of the studied glycans (Supplementary S2, Table S4). As an example, Figure 1c shows the age correlation for anti-Le^C^ IgM.

For healthy donors, moderate negative correlations (ρ = −0.49 to −0.27) were observed, that were statistically significant for all glycans (Supplementary S2, Table S5). Figure 1d exemplifies this correlation pattern for anti-Le^C^ antibodies.

### 2.3. IgG

The data on IgG levels in healthy donors and GC patients are presented in Supplementary S2, Table S6. Significant differences in AGA-IgG between GC patients and donors were only detected for oligosaccharides containing the Le^C^ in a core (Nos. 85, 267, and 299, Table 1 and Table 2), with higher levels observed in patients (Supplementary S2, Table S6).

In contrast to IgM, the AGA-IgG level showed no age-dependent correlations (Supplementary S2, Tables S7–S10).

Comparison of AGA-IgM and IgG levels between GC patients and healthy donors in the 35–50 age subgroup: Given the significant age differences between the GC patient and healthy donor cohorts (see Section 4.1 in Materials and Methods), we analyzed age-matched subgroups (35–50 years) with comparable sizes. The characteristics of these subgroups are summarized in Table 3.

The results showed that IgM levels were lower in GC patients compared with healthy donors (Supplementary S2, Table S11). The observed differences for antibodies against Galβ1-3GalNAcβ (T_ββ_) reached a significance level of 0.1 (*p* = 0.105), indicating a consistent trend toward AGA deficiency in patients.

A similar result was observed for AGA-IgG: the level was lower in GC patients than in healthy donors (Supplementary S2, Table S12). For antibodies binding to oligosaccharides containing the Le^C^ core, the *p*-values were also around 0.1: Le^C^ (*p* = 0.123), GlcNAcβ1-3Galβ1-3GlcNAcβ (*p* = 0.075), and Neu5Acα1-3Galβ1-3GlcNAcβ (*p* = 0.103).

### 2.4. AGA Interaction with Gastric Tissue

Since the surveillance function of AGAs implies their interaction with tumor cells, we investigated antibody binding to GC tissue. Anti-GalNAcα (Tn) antibodies—representing one of the best-characterized TACAs in GI cancers [4,6]—were isolated from pooled healthy donor sera (containing primarily nAbs) using hapten-specific affinity chromatography. These antibodies were tested for interaction with GC tissue and adjacent morphologically normal tissue from a patient with histologically confirmed tubular adenocarcinoma (G1–G2). Notably, the antibody concentration used (10 μg/mL) was several-fold higher than physiological level [39].

**Figure 2:**
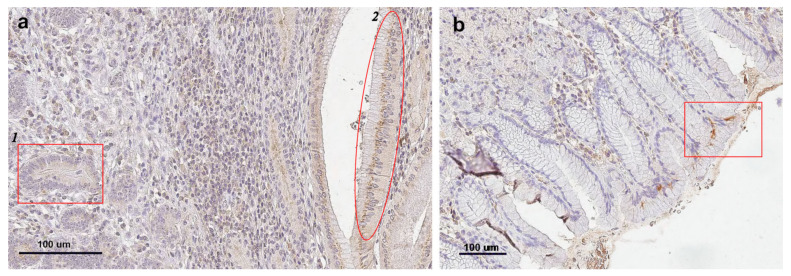
(**a**) Immunohistochemical staining of GC tissue (differentiation grade G1) with human anti-GalNAcα antibodies. 1—weak perinuclear staining of tumor gland-like structures, 2—staining in normal glands at the tumor periphery. (**b**) Immunohistochemical staining of normal gastric mucosa tissue with human anti-GalNAcα antibodies. The highlighted rectangle demonstrates membrane and perinuclear staining of mucosal surface cells.

An immunohistochemical analysis of paraffin-embedded tissue sections revealed weak perinuclear staining in tumor and gland-like structures of GC tissue, along with perinuclear and membrane staining in normal, non-tumor surface cells of healthy tissue (Figure 2a,b).

## 3. Discussion

Altered glycosylation in GI epithelial tumor cells has been suggested to result in changes in the peripheral blood antibody repertoire [18,21,40]. Owing to highly represented PGAs and cohort studies of AGA levels in cancer patients (e.g., [16,20,34]), researchers have identified antibodies not only against established TACAs (pioneered by S. Hakomori [41]), but also against other glycans, which can also be considered as tumor antigens; therefore, they were also included in the study. This group comprises carbohydrate fragments including N-chain inner-core structures and mucin disaccharide (known as core 3), along with classical TACAs having additional substituents such as sialylated Le^C^ and sulfated Tn (Table 1). The trisaccharide Galili was also investigated since human anti-Galα antibodies demonstrate binding to tumor cells and cell lines [37], though the origin of this antigen on the cells remains debated [42].

Anti-TACA antibodies hold promise not just as diagnostic markers, but also as therapeutic and preventive agents in oncology [3,8,36,43,44]. AGAs are supposed to participate in immune surveillance by identifying and eliminating emerging tumor cells [43,44]. However, aging leads to depletion of the B1-cell pool and consequent reduction in antibody production (including AGAs), potentially compromising tumor surveillance—explaining why advanced age constitutes a major cancer risk factor [22,45]. This creates a dilemma: whether a low immunoglobulin level in elderly individuals triggers oncogenesis, or whether tumor development causes this deficiency [46]. Therefore, when discussing antibody levels, we cautiously use the term “reduced” rather than “decreasing” (the latter implying longitudinal observation).

An analysis of the PGA data revealed that the antibody level (both IgG and IgM classes) against nearly all TACAs was higher in donors compared with patients. All IgM differences were statistically significant except for antibodies against Neu5Gcα (Supplementary S2, Table S1). This non-human sialic acid (xenoantigen) enters the human body through diet and is incorporated into glycoconjugates, primarily in GI organs, promoting cancer development [47,48].

For IgG, significant differences between GC patients and donors were only observed for the Le^C^ disaccharide (No. 85) and related glycans (Nos. 267 and 299) containing the Le^C^-core (Table 1 and Table 2), which serves as a common epitope for antibodies recognizing this TACA [33].

When stratifying the cohort by age groups, the highest median AGA signals were predictably found in individuals aged 20–29 years, while the lowest were in those over 60, correlating with changes in the total immunoglobulin level (as demonstrated for IgM in Figure 1 and Supplementary S2, Tables S2 and S3). However, the correlation analysis showed practically no relationship between AGA-IgM level and age in patients, whereas healthy donors exhibited moderate IgM correlations (Supplementary S2, Tables S4 and S5); also illustrated for anti-Le^C^ antibodies in Figure 1. No age-related dependence of IgG level was found (Supplementary S2, Tables S9 and S10). This finding suggests that while the TACA-specific IgM level is indeed lower in GC patients, the cohort’s age imbalance (with a significant difference between patients and donors, *p* < 0.001) prevents definitive conclusions at this stage.

To address this, we established age-matched subgroups of GC patients and donors (Table 3). A comparative analysis confirmed reduced IgM against T_ββ_ and IgG against Le^C^ and its sialylated/GlcNAcβ derivatives in GC patients, with significance reaching *p*~0.1, a threshold acceptable for pilot studies. Notably, T_ββ_ is a known TACA (Table 1), while Le^C^ (and presumably its analogs [38]) has demonstrated prognostic value in breast cancer [32,41,49,50,51].

In summary, this study revealed two key findings: (1) a reduced level of AGAs in patients versus donors, and (2) a general age-related decline in these antibodies across all individuals. While aging confounds the interpretation of the first finding, comparisons between age-matched groups support both observations. This suggests that patients with GC have an inherently insufficient level of naturally occurring AGAs—particularly against the TACAs discussed—for effective immune surveillance. This deficiency appears to be exacerbated by age.

These insights direct us toward developing therapeutic strategies aimed at replenishing such AGA deficiencies in cancer. This approach aligns well with the emerging field of “natural medicine”, which seeks to harness natural components for treatment. However, research into specific natural immunity remains limited, and is directed toward, e.g., studies on B1a-cell levels following radical surgery [52]; the clinical significance of natural monoclonal anti-glycan IgM [44,53]; the cytotoxic action of AGAs [27]; and the potential for modulating antibody levels through microbiota manipulation [54]. Detected age-related decline in antibody levels also opens up the possibility of using AGA for the diagnosis of early-stage GC.

The surveillance function obviously involves specific binding of the antibody to the target cell. The histochemical studies demonstrated binding of human anti-GalNAcα antibodies to tumor cells, which was expected since GalNAcα is a TACA in GI cancer. The interaction with anti-GalNAcα antibodies was observed in the perinuclear region—the space surrounding the nucleus that connects with the endoplasmic reticulum (ER) lumen. Normally, O-glycosylation occurs in the Golgi apparatus, where GalNAcα-transferases (GalNAc-Ts) are transported from the ER. However, as shown in [55], tumor cells exhibit disrupted GalNAc-T trafficking, leading to relocation of Tn-antigen biosynthesis to the ER, which correlates with enhanced tumor colonization.

Unexpectedly, the isolated anti-GalNAcα antibodies also interacted with morphologically normal cells surrounding the tumor and in adjacent normal tissues. Solid tumors are known to have a peritumoral zone consisting of cells from the organ of origin, but exhibiting characteristics distinct from healthy tissue [56]. This zone may extend 2–4 cm from the tumor margin [57]. In [58], comparative molecular profiling (through gene expression analysis) of three tissue types—tumor tissues (from intestine, kidney, and lung), adjacent tissues (from the same patients), and normal autopsy tissues—showed that the key differences in peritumoral cells involve their inflammatory status and disrupted intracellular signaling pathways. Specifically, these “normal” adjacent tissues exhibited, for example, altered immune cell activation (e.g., enhanced neutrophil degranulation); modified cellular respiration; cytoskeletal remodeling; extracellular matrix reorganization, along with disrupted ER-to-Golgi transport; apoptotic signaling; and cell cycle regulation compared with true normal tissues [58]. Thus, such tissues cannot be considered normal controls. Notably, the observed neutrophil degranulation in peritumoral tissues [58] releases proinflammatory cytokines that subsequently modulate glycosyltransferase expression. This alters cell surface glycoconjugate composition, affecting interactions with inflammatory mediators and the functional properties of recruited immune cells (i.e., their pro- vs. anti-inflammatory effects) [59]. For instance, Crohn’s disease (autoimmune GI inflammation) shows increased truncated glycans (Tn and sialyl-Tn—both TACAs) on intestinal mucins [60], consistent with our histochemical findings of anti-GalNAcα binding to peritumoral cells. Therefore, the hypothesis about naturally occurring AGAs surveilling tumor cells [53] should likely be expanded to include control over the tumor microenvironment, though the mechanisms remain to be elucidated.

## 4. Materials and Methods

### 4.1. Participants

The study included 235 patients with morphologically confirmed stage I–IV GC who underwent examination and treatment at the Department of Abdominal Oncology 1 of the N.N. Blokhin National Medical Research Centre of Oncology (NMRCO) from 2016 to 2018. GC staging was performed according to the 7th edition of the TNM classification system. Patients had not received prior chemo-radiotherapy. Those with multiple primary tumors or chronic inflammatory diseases in the acute phase were excluded.

Peripheral blood samples were collected from all patients before initiating anticancer therapy. Peripheral blood from healthy donors was provided by the local biobank of the NMRCO. Informed consent was obtained from all participating patients and donors. The study protocol was approved by the Local Ethics Committee of the NMRCO (Protocol No. 2, from 27 February 2025).

The characteristics of the GC patients and conditionally healthy donors are presented in Table 4 and Table 5.

A comparative analysis of GC patients and donor groups revealed statistically significant differences in age (*p* < 0.001), but not in sex (*p* = 0.092, with male predominance).

### 4.2. Sample Collection and Reagents

Blood serum samples were collected in VACUETTE^®^ tubes (Greiner Bio-One, Kremsmünster, Austria), 13 × 75 mm, 4 mL, containing a clot activator. After incubation for 1 h at +4 °C, samples were centrifuged at 2000× *g* for 10 min. The resulting serum was aliquoted and stored at −20 °C.

The study was performed using commercially available reagents (organic compounds and inorganic salts) purchased from the following manufacturers: Acros Organics (Geel, Belgium), SigmaAldrich (Saint Louis, MO, USA), Helicon (Moscow, Russia), or EKO Service (Moscow, Russia); stated if otherwise. All reagents had a minimum purity standard of analytical grade (≥99%).

### 4.3. Antibody Analysis Using PGA

A PGA containing 309 glycans (GlycoNZ LLC, Auckland, New Zealand), printed as previously described [62], was used. Glycans at 20 μM concentration were spotted onto N-hydroxysuccinimide-activated Slides H (Schott Nexterion, Jena, Germany) using a sciFLEXARRAYER S5 non-contact robot (Scienion, Berlin, Germany) at 10 replicates. Immobilization of ligands was confirmed using standard human plasma and a panel of plant lectins [62,63]. Quality control of the PGA was performed as described in [31].

For analysis, 900 μL of serum diluted 1:15 in 0.15 M phosphate-buffered saline (PBS) containing 0.1% Tween-20 (PBS-0.1%T, pH 7.4) and 1% bovine serum albumin (BSA, Sigma Aldrich, Saint Louis, MO, USA) was applied to the glycan arrays (pre-equilibrated for 15 min in PBS-0.1%T) and incubated at 37 °C with high humidity for 1 h. The arrays were washed with PBS-0.05%T and then incubated with Alexa647-labeled goat anti-human IgG (1:800 dilution in PBS-0.1%T containing 1% BSA) and Cy3-labeled anti-human IgM (1:1000 dilution in PBS-0.1%T containing 1% BSA) antibodies (Jackson Immuno Research, West Grove, PA, USA) for 1 h at 37 °C. The arrays were subsequently washed with PBS-0.05%T and deionized water, then dried by centrifugation. Fluorescence signals (expressed in relative fluorescence units, RFU, reflecting antibody level) were acquired using an InnoScan 1100 AL microarray fluorescence scanner (Innopsys, Carbonne, France) at 10 μm resolution. The obtained images were processed using the ScanArray Express 4.0 software (Perkin Elmer, Shelton, CT, USA) with fixed-circle analysis and Microsoft Excel. For subsequent statistical analysis, median values from 10 replicate measurements were used.

Only the results of antibody binding to glycans that are either established or putative TACAs according to the literature were analyzed; these are listed in Table 1 and Table 2. Complete datasets for the patients and donors are provided in Supplementary S1.

Glycans with signals below the 5th percentile of the standard deviation across replicates (i.e., low intensity) were considered insignificant and excluded from the analysis. For IgG, the threshold value was 1507 RFU, for IgM it was 1652 RFU.

### 4.4. Quantitative Analysis of Total Immunoglobulins

Mouse anti-human IgM antibodies (Southern Biotech, Birmingham, AL, USA) diluted 1:200 in 0.05 M sodium carbonate buffer (pH 9.6) were added to wells of 96-well polystyrene plates (Stripwell Microplate, high binding, Costar, Kennebunk, ME, USA) at 100 μL/well. Immobilization was performed for 1 h at 37 °C. The wells were blocked with PBS containing 1% BSA at 120 μL/well for 1 h at 37 °C. After blocking, plate wells were washed 3 times with PBS-0.1%T at 160 μL/well. Pooled serum samples (from 11 randomly selected donors/patients belonging to each subgroup) were diluted 3625-fold based on literature data on the average immunoglobulin content in human blood (14.5 mg/mL [64]) to achieve a final antibody concentration of ~4 μg/mL per well. Diluted samples were added to the first plate wells (in duplicate). As a control, human IgM standards (Southern Biotech, Birmingham, AL, USA) with an initial concentration of 0.2 μg/mL at 100 μL/well were used. The samples were subjected to six sequential two-fold dilutions.

The plate was incubated for 1 h at 37 °C, after which the wells were washed four times with PBS-0.1%T at 160 μL/well. Then, secondary antibodies against human Ig(G + M + A) conjugated with horseradish peroxidase (Southern Biotech, Birmingham, AL, USA) at 1:2500 dilution were added at 100 μL/well and incubated for 1 h at 37 °C, followed by four washes with PBS-0.1%T at 160 μL/well. To detect activity of antibody-bound horseradish peroxidase, 100 μL of 0.075 M phosphate-citrate buffer (pH 5.0) with 0.04% o-phenylenediamine and 0.03% H_2_O_2_ was added to the plate wells, followed by incubation for 15 min at room temperature. The color reaction was stopped by adding 1 M H_2_SO_4_ at 30 μL/well. Optical density (OD) was measured at 490 nm using a Wallac 1420 Multilabel Counter plate reader (Perkin Elmer, Shelton, CT, USA). The obtained data were processed using Microsoft Excel 2013 and the Origin 7.0 software by constructing a calibration curve using data from the human IgM standard (ThermoFisher, Waltham, MA, USA) of known concentration.

### 4.5. Isolation of Human Anti-GalNAcα Antibodies

Antibodies were isolated from pooled sera of conditionally healthy donors (the pool included 64 serum samples from donors, regardless of sex and ABO blood group) using affinity chromatography on GalNAcα-Sepharose 6FF sorbent (Synthaur LLC, Moscow, Russia), and their specificity was confirmed as previously described [39].

### 4.6. Immunohistochemistry

Postoperative samples from a 74-year-old patient with the proximal stomach carcinoma cT4aN2M0 (cyt-), stage III, and adjacent healthy tissue obtained after gastrectomy performed at the NMRCO, Moscow, were fixed in buffered 4% formaldehyde and embedded in paraffin. Tissue sections of 2 μm thickness were deparaffinized with xylene and rehydrated. Deparaffinized sections underwent antigen unmasking process at 95 °C (30 min) in R-UNIVERSAL buffer (Aptum Biologics Ltd., Southampton, UK). After blocking endogenous peroxidase activity, 100 μL of anti-GalNAcα antibodies (isolated as described above) at a concentration of 10 μg/mL in PBS were applied and incubated overnight at +4 °C. After PBS washing, for visualization of bound antibodies in tissue structures, the sections were incubated with 100 μL of mouse antibodies against human IgM and IgG conjugated with horseradish peroxidase (Southern Biotech, Birmingham, AL, USA), then diluted 1:500 in PBS, followed by application of the DAB Peroxidase Substrate Kit detection system according to the manufacturer’s protocol (Vector Laboratories, Newark, CA, USA). Nuclei were counterstained with Mayer’s hematoxylin according to the manufacturer’s protocol (Biovitrum, Moscow, Russia), with subsequent mounting of sections in permanent mounting medium. The stained sections were examined on a motorized ZEISS Axio Imager.Z2 microscope using a Zeiss alpha Plan-Apochrom 100×/1.46 Oil DIC M27 objective (Carl Zeiss Vision, Aalen, Germany). The obtained images were processed using the ZEN Module Bundle Intellesis & Analysis for Light Microscopy software, version 3.4.91.00000 (Carl Zeiss Vision, Aalen, Germany).

### 4.7. Statistical Analysis

Statistical analysis was performed using the StatTech v. 4.0.7 software (StatTech LLC, Moscow, Russia) and SPSS version 21.0 (IBM, Armonk, NY, USA). Quantitative variables were assessed for normal distribution using the Shapiro–Wilk test (for sample sizes < 50) or the Kolmogorov–Smirnov test (for sample sizes > 50). When normal distribution was absent, quantitative data were described using the median (Me) of 10 ligand replicates on the PGA with interquartile range (Q1–Q3). Comparison of three or more groups for quantitative variables with non-normal distribution was performed using the Kruskal–Wallis test, with post hoc comparisons conducted using Dunn’s test with Holm’s correction for multiple comparisons. The direction and strength of correlation between two quantitative variables were assessed using Spearman’s rank correlation coefficient (for non-normally distributed variables).

## 5. Conclusions

In gastric cancer treatment, both surgical and chemotherapeutic approaches have largely reached their limits of effectiveness, while the use of monoclonal antibodies remains limited. Therefore, the search for new strategies is necessary. The study presented here reveals that decreased levels of antibodies to TACAs indicate a risk of developing this disease. Combined with existing knowledge on the origin of naturally occurring AGAs, their cytotoxic effect, and the ability to specifically modulate their levels using microbiota, the data described here allow the formulation of a “natural medicine” strategy—an approach which relies on the innate immune system; specifically, the targeted replenishment of AGAs that function to prevent cancer development.

## Figures and Tables

**Table 1:** Structures and characteristics of tumor-associated glycans.

Glycan ID	Glycan Structure (General Name)	SNFG Representation ^1^	Glycan Characteristics	Potential Clinical Applications
4	GalNAcα1-O-Ser (Tn-Ser ^2^)	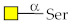	Core fragment of complex mucin glycans. Involved in adhesion and invasion.	Diagnosis, therapeutic target, prognosis
5	GalNAcα (Tn)	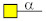	Analog of Tn.	Diagnosis, therapeutic target, prognosis
52	Neu5Gcα	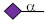	Xenogenic antigen acquired through diet (red meat) as part of complex glycoconjugates. Hydrolyzed to monosaccharides in cells and incorporated into human glycoconjugates via CMP derivatives, competing with Neu5Ac.	Diagnosis, therapeutic target
78	Galα1-3GalNAcα (T_αα_)	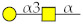	Core structure of bronchial mucin glycans (teratocarcinoma cells).	Diagnosis, therapeutic target
85	Galβ1-3GlcNAcβ (Le^C^)	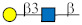	Terminal fragment of N-glycan chains. Identified in own studies.	Prognosis, therapy, prevention
88	Galβ1-3GalNAcβ (T_ββ_)	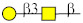	Terminal part of ganglio-series glycosphingolipid asialo-GM1.	Diagnosis
89	Galβ1-3GalNAcα (TF)	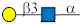	Core part of mucin glycans.	Anti-TF antibody level may refine diagnostic accuracy
172	Neu5Acα2-6GalNAcα (SiaTn)	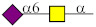	Disaccharide component of mucin glycans.	Diagnosis
174	Neu5Gcα2-6GalNAcα (Neu5Gc-Tn)	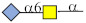	Disaccharide component of mucin glycans (see no. 52).	Under investigation
223	Galα1-4Galβ1-4Glcβ (P^k^)	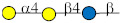	Glycan of blood group P system.	Diagnosis, therapeutic target
254	GlcNAcβ1- 6(Galβ1-3)GalNAcα (core 2)	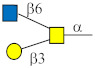	Core fragment of mucin glycans.	Under investigation
267	GlcNAcβ1-3Galβ1- 3GlcNAcβ (GlcNAcβ3’Le^C^)	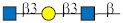	Analog of Le^C^ [32,33].	Under investigation

^1^ Glycan structures (here and elsewhere) are presented according to the Symbol Nomenclature for Glycans (SNFG, URL: https://www.ncbi.nlm.nih.gov/glycans/snfg.html (accessed on 8 January 2026)). ^2^ Serine or threonine.

**Table 2:** Structures of glycans considered as TACAs and their characteristics.

Glycan ID	Glycan Structure (General Name)	SNFG Representation ^1^	Glycan Characteristics	Potential Clinical Applications
17	Manα-Gly ^1^	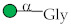	Identified in our studies [34].	Diagnosis, therapeutic target
18	Manβ-Gly	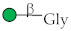	Identified in our studies [34].	Diagnosis, therapeutic target
36	Manβ	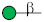	Analog of Manβ-Gly (no. 18; see above).	Diagnosis, therapeutic target
41	6-O-Su-GalNAcα ^2^	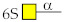	Identified in our studies [34].	Under investigation
113	GlcNAcβ1-3GalNAcα (core 3)	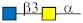	Core structure of mucin glycans. Selected based on literature data [35].	Under investigation
114	GlcNAcβ1-3Manβ-Gly	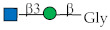	Core fragment of N-glycan chains. Antibodies detected in our studies [19].	Under investigation
122	Manα1-6Manβ-Gly	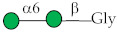	Core fragment of N-glycan chains. Selected in our studies [34].	Under investigation
123	Manβ1-4GlcNAcβ-Gly	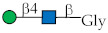	Core fragment of N-glycan chains. Selected based on literature data [36].	Diagnosis
222	Galα1-3Galβ1-4GlcNAcβ (Galili)	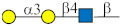	Galili xenoantigen [37].	Therapeutic target
258	(Manα1)_2_-3,6Manβ-Gly	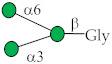	Core fragment of N-glycan chains [34].	Under investigation
299	Neu5Acα2-3Galβ1-3GlcNAcβ (3’SiaLe^C^)	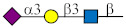	Terminal fragment of N-glycan chains. Selected based on our studies [33,38].	Under investigation

^1^ Gly—glycyl spacer (-NHCOCH_2_NH_2_). ^2^ Su or S—sulfate residue.

**Table 3:** Demographic and clinical characteristics of GC patients and healthy donors in the 35–50 age subgroup.

Parameter	GC Patients	Healthy Donors
**Age, years** (Median (Q1–Q3))	43.00 (38.50–48.00)	39.50 (37.25–43.00)
**Number of subjects**	31	30
*p*	0.061	
**Gender**		
Male (*n* (%))	16 (51.6)	24 (80)
Female (*n* (%))	15 (48.4)	6 (20)
**Disease Stage**		
Stage I (*n* (%))	3 (9.7)	−
Stage II (*n* (%))	7 (22.6)	−
Stage III (*n* (%))	9 (29.0)	−
Stage IV (*n* (%))	12 (38.7)	−

**Table 4:** Characteristics of GC patients (*n* = 235).

Parameter	Category	Number in Group	Percentage of Patients, %
**Age**	Median: 61 years (range: 27–80)		
**Gender**	Male	125	53.2
	Female	110	46.8
**Age groups**	20–29 years	4	1.7
	30–39 years	11	4.7
	40–49 years	20	8.5
	50–59 years	65	27.7
	60–69 years	83	35.3
	70–80 years	52	22.1
**Stage**	Stage I	37	15.7
	Stage II	59	25.1
	Stage III	77	32.8
	Stage IV	62	26.4
**T category**	T1	41	17.4
	T2	12	5.1
	T3	42	17.9
	T4	140	59.6
**N category** **(assessed in 209 patients)**	N0	89	42.6
	N1	45	21.5
	N2	39	18.7
	N3	36	17.2
**M category**	M0	183	77.9
	M1	52	22.1
**Lauren Classification** [61] **(assessed in 112 patients)**	Intestinal type	54	48.2
	Diffuse type	51	45.5
	Mixed type	7	6.2
**WHO histological type**	Well-differentiated adenocarcinoma	8	3.4
	Moderately differentiated adenocarcinoma	70	29.8
	Poorly differentiated adenocarcinoma	68	28.9
	Signet-ring cell carcinoma	53	22.6
	Mixed type	36	15.3

**Table 5:** Characteristics of healthy donors (*n* = 76).

Parameter	Category	Number in Group	Percentage of Donors, %
**Age**	Median—34 years (range: 21–56)		
**Gender**	Male	49	64.5
	Female	27	35.5
**Age Groups**	20–29 years	25	32.9
	30–39 years	32	42.1
	40–49 years	15	19.7
	50–59 years	4	5.3

## Data Availability

The original contributions presented in this study are included in the Supplementary Material. Further inquiries can be directed to the corresponding author.

## Supplementary Materials

The following supporting information can be downloaded from https://www.mdpi.com/article/10.3390/ijms27020800/s1.

## Abbreviations

The following abbreviations are used in this manuscript:

GC	Gastric cancer
TACAs	Tumor-associated carbohydrate antigens
PGA	Printed glycan array
nAbs	Naturally occurring antibodies
GI	Gastrointestinal
AGA	Anti-glycan antibody
NMRCO	N.N. Blokhin National Medical Research Centre of Oncology
RFUs	Relative fluorescence units
ER	Endoplasmic reticulum
