# Supplementary material for: The Level of Antibodies to Tumor-Associated Glycans in Gastric Cancer Patients Is Lower than in Healthy Donors and Reduces with Age
Source: Int J Mol Sci. 2026 Jan 13;27(2):800. doi: 10.3390/ijms27020800 (PMC12841265; doi:10.3390/ijms27020800)
Supplement: Supplementary file 1 [file ijms-27-00800-s001.zip › Supplementary S2_Article_Nikulin.pdf]

Supplementary material 2

The level of antibodies to tumor-associated glycans in gastric cancer patients is lower than in healthy donors and reduces with age

Maxim P. Nikulin[1*#], Alexander D. Lipatnikov[2], Alexei Yu. Nokel[3], Svetlana M. Polyakova[2], Svetlana V. Tsygankova[2], Galina V. Pazynina[2], Alexandra V. Semyanikhina[1,4], Elena V. Ogorodnikova[1], Dmitry V. Rogozhin[1], Oxana M. Rossomakhina[5], Dmitry A. Atyakshin[6], Olga I. Patsap[6], Ivan S. Stilidi[1], Nikolai V. Bovin[2#], Igor Buchwalow[6,7], Markus Tiemann[7] and Nadezhda V. Shilova[2,3#]

[1] Federal State Budgetary Institution "National Medical Research Center of Oncology named after N.N. Blokhin" of the Ministry of Health of the Russian Federation, 23, Kashirskoe shosse, Moscow 115522 Russia;

[2]Shemyakin-Ovchinnikov Institute of Bioorganic Chemistry of the Russian Academy of Sciences, 16/10, Ulitsa Miklukho-Maklaya Moscow 117997, Russia;

[3]Federal State Budgetary Institution "National Medical Research Center for Obstetrics, Gynecology and Perinatology named after Academician V.I. Kulakov" of the Ministry of Health of the Russian Federation, 4, Oparina street, Moscow 117997, Russia;

[4]Federal State Budgetary Scientific Institution Research Centre of Medical Genetics, 1, Moskvorechye St,
Moscow 115522 Russia;

[5]Federal State Budgetary Educational Institution of Higher Education "Lugansk State Medical University named after St. Luke" of the Ministry of Health of the Russian Federation, 1G, quarter of the 50th anniversary of the Defense of Lugansk, Lugansk 291045, Russia;

[6]Research and Educational Resource Center for Immunophenotyping, Digital Spatial Profiling and Ultrastructural Analysis Innovative Technologies, Peoples' Friendship University of Russia, 6, Miklukho-Maklaya str., Moscow 117198, Russia;

[7]Institute for Hematopathology, Fangdieckstr., 75a, 22547 Hamburg, Germany.

*Correspondence: Maxim P. Nikulin maximpetrovich@mail.ru Tel.: +7 903 16-31-88

[#] These authors contributed equally to this work

Table S1. The comparison of anti-glycan antibody~~ies~~ level* (IgM) in blood of gastric cancer patients and healthy donors.

Glycan ID	Glycan (trivial name)	Patients (n=235)			Donors (n=76)			*p*
		Me**	Q_1	Q_3	Me	Q_1	Q_3	
4	GalNAcα1-O-Ser (Tn-Ser)	1998	901	3817	3088	1714	6498	<0,001***
5	GalNAcα (Tn)	3068	1299	5462	5079	2098	8615	<0,001*
17	Manα-Gly	624	340	1173	1051	568	1588	<0,001*
18	Manβ-Gly	533	278	1062	963	512	1519	<0,001*
36	Manβ-C3	2958	1019	7320	5834	2608	11170	0,002*
41	6-O-Su-GalNAcα	6571	3061	10869	7818	3661	15971	0,016
52	Neu5Gcα	565	272	1114	724	408	1228	0,072
78	Galα1-3GalNAcα ($T_{αα}$)	3196	1124	9429	9301	4436	19510	<0,001*
85	Galβ1-3GlcNAcβ (Le^C)	3496	1873	8701	9012	4965	20796	<0,001*
88	Galβ1-3GalNAcβ ($T_{ββ}$)	1498	570	3974	3576	1774	8577	<0,001*
89	Galβ1-3GalNAcα (TF)	4497	1952	9042	8569	3687	19662	<0,001*
113	GlcNAcβ1-3GalNAcα (core 3)	917	429	2533	1795	757	4137	<0,001*
114	GlcNAcβ1-3Manβ-Gly	498	258	1066	770	390	1268	<0,001*
122	Manα1-6Manβ-Gly	3841	1692	9542	6438	2718	11882	0,006*
123	Manβ1-4GlcNAcβ-Gly	2086	780	4306	3046	1215	7552	0,010*
172	Neu5Acα2-6GalNAcα (SiaTn)	2530	954	5077	3528	1605	7884	0,003*
174	Neu5Gcα2-6GalNAcα (Neu5GcTn)	2737	1341	5304	5339	2739	10020	0,006*
222	Galα1-3Galβ1-4GlcNAcβ (Galili)	3604	1514	6747	6381	3104	13800	<0,001*
223	Galα1-4Galβ1-4Glcβ (P_k)	1189	384	3304	2950	1416	7665	<0,001*
254	GlcNAcβ1-6(Galβ1-3) GalNAcα (core 2)	600	303	1071	910	467	1542	<0,001*
258	$(Manα1)_2$-3,6Manβ-Gly	2962	1143	8319	8932	4439	17797	0,001*
267	GlcNAcβ1-3Galβ1-3GlcNAcβ (GlcNAcβ3′Le^C)	3304	1282	9175	10464	4495	19021	<0,001*
299	Neu5Acα2-3Galβ1-3GlcNAcβ (3′SiaLe^C)	1998	901	3817	3088	1714	6498	<0,001*

* Here and hereafter, ~~—~~the level of anti-glycan antibodies is expressed in relative fluorescent units (RFUs).

** Here and hereafter, ~~—~~Me: ~~e—~~median of RFU; ~~,~~ Q1–~~—~~Q3: ~~-~~interquartile range.

*** The difference is statistically significant ($p < 0,05$).

Table S2. The level of anti-glycan antibodies (IgM) in blood of gastric cancer patients in different age groups

Glycan ID	Glycan (trivial name)	Age group	Antibody levels			*p*
			Me	$Q_1 – Q_3$	n	
4	GalNAcα1-O-Ser (Tn-Ser)	20-29	4803	2013 – 7937	4	0,149
		30-39	2370	1843– 7626	11	
		40-49	2239	766 – 4030	20	
		50-59	2074	1153 – 4434	65	
		60-69	1628	675 – 3214	83	
		70-80	1823	869 – 3343	52	
5	GalNAcα (Tn)	20-29	6716	4891 – 9988	4	0,245
		30-39	4384	2474 – 6309	11	
		40-49	2379	1264 – 4588	20	
		50-59	3389	1469– 5895	65	
		60-69	2599	1204 – 5192	83	
		70-80	2133	1358 – 4446	52	
17	Manα-Gly	20-29	1055	853 – 1478	4	0,201
		30-39	800	574 – 1405	11	
		40-49	739	361 – 1105	20	
		50-59	677	383 – 1159	65	
		60-69	494	268 – 1124	83	
		70-80	480	333 – 1120	52	
18	Manβ-Gly	20-29	922	743 – 1354	4	0,086
		30-39	696	549 – 1419	11	
		40-49	797	329 – 1020	20	
		50-59	618	388 – 1233	65	
		60-69	446	224 – 970	83	
		70-80	444	246 – 995	52	
36		20-29	6834	2798 – 10191	4	0,266

	Manβ-C3	30-39	2947	1824 – 7581	11	
		40-49	3598	1168 – 9832	20	
		50-59	4225	1507 – 7320	65	
		60-69	2372	689 – 6258	83	
		70-80	1953	1051 – 6456	52	
41	6-*O*-Su-GalNAcα	20-29	16927	13410 – 20300	4	0,008*
		30-39	9866	5371 – 16092	11	
		40-49	5893	4454 – 9166	20	
		50-59	7194	3490 – 11853	65	
		60-69	4285	2512 – 9071	83	
		70-80	5300	3442 – 10380	52	
52	Neu5Gcα	20-29	1395	582 – 2261	4	0,084
		30-39	1072	564 – 1788	11	
		40-49	788	255 – 1388	20	
		50-59	652	289 – 1292	65	
		60-69	503	222 – 88	83	
		70-80	496	281 – 791	52	
75	Galα1-3Galβ	20-29	6739	1526 – 12575	4	0,140
		30-39	7461	3291 – 13055	11	
		40-49	3775	1933 – 7025	20	
		50-59	4966	1851 – 9027	65	
		60-69	3693	1613 – 6311	83	
		70-80	3184	1364 – 59201	52	
78	Galα1-3GalNAcα ($T_{\alpha\alpha}$)	20-29	2181	1452 – 3752	4	0,049* $p_{60-69 – 40-49}$ = 0,024
		30-39	2452	1114 – 6267	11	
		40-49	4252	2083 – 8896	20	
		50-59	1323	656 – 4937	65	
		60-69	1460	547 – 3919	83	
		70-80	1483	595 – 3752	52	
85	Galβ1-3GlcNAcβ (Le^C)	20-29	6574	4984 – 9254	4	0,009*
		30-39	7562	2814 – 14247	11	

		40-49	6639	1494 – 13752	20	
		50-59	4903	1925 – 10129	65	
		60-69	2675	1026 – 6013	83	
		70-80	1842	851 – 7313	52	
88	Galβ1-3GalNAcβ ($T_{\beta\beta}$)	20-29	6089	4321 – 11334	4	0,006* $p_{60-69 – 50-59}$ = 0,024
		30-39	5280	3697 – 15066	11	
		40-49	3833	2150 – 9714	20	
		50-59	4622	2463 – 12456	65	
		60-69	2610	1229 – 5631	83	
		70-80	3083	1756 – 7801	52	
89	Galβ1-3GalNAcα (TF)	20-29	4458	1402 – 7808	4	0,088
		30-39	1595	1232 – 4454	11	
		40-49	2546	1188 – 3560	20	
		50-59	1337	630 – 3974	65	
		60-69	840	447 – 3175	83	
		70-80	1545	4967 – 4300	52	
113	GlcNAcβ1-3GalNAcα (core 3)	20-29	9917	8018 – 13348	4	0,007* $p_{60-69 – 30-39}$ = 0,026
		30-39	9236	6376 – 14089	11	
		40-49	4967	2713 – 7818	20	
		50-59	4200	2304 – 10836	65	
		60-69	3757	1261 – 7209	83	
		70-80	3740	1674 – 9048	52	
114	GlcNAcβ1-3Manβ-Gly	20-29	2385	1910 – 3169	4	0,314
		30-39	835	752 – 2320	11	
		40-49	1409	388 – 3910	20	
		50-59	937	564 – 2571	65	
		60-69	943	366 – 2154	83	
		70-80	607	367 – 1619	52	
122	Manα1-6Manβ-Gly	20-29	1238	880 – 2337	4	0,119
		30-39	1071	520 – 1334	11	
		40-49	648	393 – 1042	20	

		50-59	494	286 – 1043	65	
		60-69	467	214 – 970	83	
		70-80	398	244 – 843	52	
123	Manβ1-4GlcNAcβ-Gly	20-29	5866	3458 – 11577	4	0,103
		30-39	9529	2855 – 10910	11	
		40-49	5665	2214 – 12162	20	
		50-59	4885	1991 – 11044	65	
		60-69	3746	1038 – 7239	83	
		70-80	3272	1822 – 6971	52	
172	Neu5Acα2-6GalNAcα (SiaTn)	20-29	6161	5135 – 6383	4	0,152
		30-39	3082	1330 – 5315	11	
		40-49	2525	836 – 4635	20	
		50-59	2377	647 – 4168	65	
		60-69	1617	621 – 3975	83	
		70-80	1615	717 – 3157	52	
174	Neu5Gcα2-6GalNAcα (Neu5GcTn)	20-29	7689	5813 – 8637	4	0,205
		30-39	3418	1561 – 6385	11	
		40-49	2408	1031 – 4925	20	
		50-59	3141	798 – 5032	65	
		60-69	2156	929 – 4957	83	
		70-80	2208	944 – 3882	52	
222	Galα1-3Galβ1-4GlcNAcβ (Galili)	20-29	4030	3963 – 4299	4	0,065
		30-39	3507	1625 – 6856	11	
		40-49	3549	2001 – 8672	20	
		50-59	2935	1671 – 6353	65	
		60-69	2084	1055 – 3974	83	
		70-80	2536	1065 – 4814	52	
223	Galα1-4Galβ1-4Glcβ (P_k)	20-29	4785	3184 – 6556	4	0,146
		30-39	4673	4151 – 11644	11	
		40-49	5984	2320 – 7557	20	
		50-59	4175	1842 – 7520	65	

		60-69	3081	1132 – 6323	83	
		70-80	3505	1469 – 5333	52	
254	GlcNAcβ1-6(Galβ1-3) GalNAcα (core 2)	20-29	3541	1080 – 6190	4	0,003*
		30-39	2065	1435 – 5764	11	
		40-49	2055	1168 – 5394	20	
		50-59	1933	458 – 3390	65	
		60-69	842	289 – 2337	83	
		70-80	743	367 – 2212	52	
258	(Manα1)$_2$-3,6Manβ-Gly	20-29	1072	815 – 1544	4	0,070
		30-39	798	586 – 1678	11	
		40-49	855	376,75 – 981	20	
		50-59	669	382 – 1139	65	
		60-69	445	238 – 973	83	
		70-80	460	243 – 1013	52	
267	GlcNAcβ1-3Galβ1-3GlcNAcβ (GlcNAcβ3'Le^C)	20-29	6256	4962 – 9147	4	0,005* $p_{70\text{-}80 – 50\text{-}59}$ = 0,048
		30-39	7806	2239 – 10821	11	
		40-49	5220	1557 – 12773	20	
		50-59	4670	1840 – 11340	65	
		60-69	2334	1042 – 5539	83	
		70-80	1715	836 – 7241	52	
299	Neu5Acα2-3Galβ1- 3GlcNAcβ (3'SiaLe^C)	20-29	7125	6156 – 9645	4	0,005*
		30-39	7098	3375 – 15436	11	
		40-49	5116	1595 – 22205	20	
		50-59	4186	1847 – 11361	65	
		60-69	3085	974 – 6912	83	
		70-80	1753	745 – 5912	52	

* The difference is statistically significant ($p < 0,05$).

Table S3. The level of anti-glycan antibodies (IgM) in blood of healthy donors in different age groups.

Glycan ID	Glycan (trivial name)	Age group	Antibody levels			p
			Me	$Q_1 - Q_3$	n	
4	GalNAcα-O-Ser (Tn-Ser)	20-29	4248	2597 – 6516	24	0,004*
		30-39	3384	1939 – 8178	33	$p_{50-59 – 20-29}$ =
		40-49	1827	853 – 3899	15	0,016
		50-59	888	482– 1309	4	$p_{50-59 – 30-39}$ = 0,026
5	GalNAcα (Tn)	20-29	5644	3468 – 10497	24	0,008*
		30-39	4698	2038 – 8618	33	$p_{50-59 – 20-29}$ =
		40-49	5258	2384 – 6797	15	0,005
		50-59	561	483 – 977	4	$p_{50-59 – 30-39}$ = 0,030
17	Manα-Gly	20-29	1339	1081 – 2194	24	0,002*
		30-39	987	598 – 1550	33	$p_{50-59 – 20-29}$ =
		40-49	752	443 – 1182	15	0,003
		50-59	351	246 – 438	4	
18	Manβ-Gly	20-29	1266	917 – 2733	24	0,002*
		30-39	1005	488 – 1413	33	$p_{50-59 – 20-29}$ =
		40-49	550	470 – 1138	15	0,002
		50-59	231	206 – 277	4	$p_{50-59 – 30-39}$ = 0,050
36	Manβ-C3	20-29	7880	4408 – 14042	24	0,005*
		30-39	6160	3584 – 10699	33	$p_{50-59 – 20-29}$ =
		40-49	5224	1207 – 7877	15	0,004
		50-59	487	3489– 598	4	$p_{50-59 – 30-39}$ = 0,014
41	6-*O*-Su-GalNAcα	20-29	12540	6204 – 17357	24	0,008*
		30-39	7802	3774 – 17511	33	$p_{50-59 – 20-29}$ =
		40-49	6418	3377 – 10572	15	0,014
		50-59	2261	1266 – 3458	4	

52	Neu5Gcα	20-29	843	646 – 1590	24	< 0,001* $p_{40-49-20-29}$ = 0,010 $p_{50-59-20-29}$ = 0,013 $p_{40-49-30-39}$ = 0,042 $p_{50-59-30-39}$ = 0,041
		30-39	825	510 – 1252	33	
		40-49	431	290 – 612	15	
		50-59	290	236 – 332	4	
75	Galα1-2Galβ	20-29	9618	6654 – 15662	24	0,007* $p_{40-49-20-29}$ = 0,010
		30-39	5711	3158 – 9950	33	
		40-49	3310	1296 – 8266	15	
		50-59	2944	592 – 6958	4	
78	Galα1-3GalNAcα ($T_{\alpha\alpha}$)	20-29	5816	3168 – 11312	24	< 0,001* $p_{40-49-20-29}$ = 0,013 $p_{50-59-20-29}$ = 0,002 $p_{50-59-30-39}$ = 0,013
		30-39	4780	1824 – 7514	33	
		40-49	2733	1164 – 4103	15	
		50-59	374	280 – 459	4	
85	Galβ1-3GlcNAcβ (Le^{C})	20-29	13863	8908 – 23995	24	0,007* $p_{40-49-20-29}$ = 0,011
		30-39	9278	4587 – 20670	33	
		40-49	5553	1908 – 10160	15	
		50-59	1792	1041 – 9243	4	
88	Galβ1-3GalNAcβ ($T_{\beta\beta}$)	20-29	12602	6569 – 30367	24	0,007* $p_{50-59-20-29}$ = 0,005 $p_{50-59-30-39}$ = 0,021
		30-39	9101	4978 – 22272	33	
		40-49	6318	5313 – 10694	15	
		50-59	1305	1125 – 1887	4	
89	Galβ1-3GalNAcα (TF)	20-29	4633	3158 – 8136	24	0,004* $p_{50-59-20-29}$ = 0,006
		30-39	3796	2066 – 10503	33	
		40-49	2760	821 – 5893	15	

		50-59	319	247 – 409	4	$p_{50-59 – 30-39}$ = 0,007
113	GlcNAcβ1-3GalNAcα (core 3)	20-29	10663	8028 – 19691	24	< 0,001* $p_{40-49 – 20-29}$ = 0,021 $p_{50-59 – 20-29}$ = 0,004 $p_{50-59 – 30-39}$ = 0,010
		30-39	10623	4732 – 23389	33	
		40-49	5273	2234 – 7622	15	
		50-59	798	438 – 1544	4	
114	GlcNAcβ1-3Manβ-Gly	20-29	2873	1738 – 4798	24	0,001* $p_{40-49 – 20-29}$ = 0,023 $p_{50-59 – 20-29}$ = 0,005 $p_{50-59 – 30-39}$ = 0,025
		30-39	1769	1167 – 3925	33	
		40-49	789	635 – 1798	15	
		50-59	507	322 – 657	4	
122	Manα1-6Manβ-Gly	20-29	1145	856 – 2293	24	< 0,001* $p_{30-39 – 20-29}$ = 0,041 $p_{40-49 – 20-29}$ = 0,007 $p_{50-59 – 20-29}$ = 0,005
		30-39	750	388 – 1095	33	
		40-49	466	328 – 865	15	
		50-59	279	211 – 351	4	
123	Manβ1-4GlcNAcβ-Gly	20-29	8376	5137 – 12709	24	0,002* $p_{50-59 – 20-29}$ = 0,004 $p_{50-59 – 30-39}$ = 0,012
		30-39	6791	2864 – 14935	33	
		40-49	4829	1689 – 6656	15	
		50-59	471	370 – 914	4	
172	Neu5Acα2-6GalNAcα (SiaTn)	20-29	4281	1858 – 8130	24	0,022* $p_{50-59 – 20-29}$ = 0,029
		30-39	4086	1408 – 9156	33	
		40-49	2415	1441 – 3519	15	
		50-59	359	274 – 610	4	

						$p_{50-59\,–\,30-39}$ = 0,029
174	Neu5Gcα2-6GalNAcα (Neu5GcTn)	20-29	5295	2029 – 9486	24	0,017*
		30-39	4632	1703 – 8514	33	$p_{50-59\,–\,20-29}$ = 0,023
		40-49	2904	2093 – 3666	15	
		50-59	480	357 – 827	4	$p_{50-59\,–\,30-39}$ = 0,025
222	Galα1-3Galβ1-4GlcNAcβ (Galilli)	20-29	8066	4688 – 12724	24	< 0,001*
		30-39	6695	35123 – 10695	33	$p_{40-49\,–\,20-29}$ = 0,007
		40-49	3027	979 – 4991	15	
		50-59	303	289 – 424	4	$p_{50-59\,–\,20-29}$ = 0,001 $p_{50-59\,–\,30-39}$ = 0,007
223	Galα1-4Galβ1-4Glcβ (P_k)	20-29	6643	3704 – 1644	24	0,017*
		30-39	8533	3997 – 13951	33	
		40-49	3467	1623 – 7836	15	
		50-59	1788	1178 – 2828	4	
254	GlcNAcβ1-6(Galβ1-3) GalNAcα (core 2)	20-29	4149	2258 – 8791	24	0,002*
		30-39	3440	2144 – 9524	33	$p_{50-59\,–\,20-29}$ = 0,005
		40-49	2005	526 – 4804	15	
		50-59	159	106 – 275	4	$p_{50-59\,–\,30-39}$ = 0,008
258	(Manα1)$_2$-3,6Manβ-Gly	20-29	1300	860 – 2546	24	< 0,001*
		30-39	971	458 – 1545	33	$p_{40-49\,–\,20-29}$ = 0,015
		40-49	605	398 – 870	15	
		50-59	237	192 – 282	4	$p_{50-59\,–\,20-29}$ = 0,002 $p_{50-59\,–\,30-39}$ = 0,025
267		20-29	11837	8647 – 22785	24	
		30-39	7467	4420 – 18304	33	

	GlcNAcβ1-3Galβ1-3GlcNAcβ (GlcNAcβ3′Le[C])	40-49	5368	2294 – 9638	15	0,006* $p_{40\text{-}49 – 20\text{-}29}$ = 0,011
		50-59	1368	883 – 8694	4	
299	Neu5Acα2-3Galβ1-3GlcNAcβ (3′SiaLe[C])	20-29	14574	9562 – 26170	24	0,003* $p_{40\text{-}49 – 20\text{-}29}$ = 0,005
		30-39	10236	4604 – 23055	33	
		40-49	5701	2506 – 10431	15	
		50-59	1858	1157 – 9545	4	

* The difference is statistically significant ($p < 0,05$).

Table S4. The results of the correlation analysis between the levels of anti-glycan antibodies (IgM) and the age of gastric cancer patients.

Glycan ID	Glycan (trivial name)	Significance of the correlation		
		ϱ	Chaddock's Scale of Association	*p*
4	GalNAcα-O-Ser (Tn-Ser)	-0,099	Negligible	0,128
5	GalNAcα (Tn)	-0,103	Weak	0,115
17	Manα-Gly	-0,095	Negligible	0,145
18	Manβ-Gly	-0,134	Weak	0,040*
36	Manβ-C3	-0,088	Negligible	0,179
41	6-*O*-Su-GalNAcα	-0,137	Weak	0,036*
52	Neu5Gcα	-0,130	Weak	0,047*
75	Gala1-2Galb	-0,103	Weak	0,116
78	Galα1-3GalNAcα ($T_{\alpha\alpha}$)	-0,097	Negligible	0,139
85	Galβ1-3GlcNAcβ (Le^{C})	-0,198	Weak	0,002*
88	Galβ1-3GalNAcβ ($T_{\beta\beta}$)	-0,156	Weak	0,017*
89	Galβ1-3GalNAcα (TF)	-0,115	Weak	0,077
113	GlcNAcβ1-3GalNAcα (core 3)	-0,146	Weak	0,025*
114	GlcNAcβ1-3Manβ-Gly	-0,123	Weak	0,059
122	Manα1-6Manβ-Gly	-0,101	Weak	0,124
123	Manβ1-4GlcNAcβ-Gly	-0,164	Weak	0,012*
172	Neu5Acα2-6GalNAcα (SiaTn)	-0,121	Weak	0,065
174	Neu5Gcα2-6GalNAcα (Neu5GcTn)	-0,102	Weak	0,118
222	Galα1-3Galβ1-4GlcNAcβ (Galilli)	-0,135	Weak	0,039*
223	Galα1-4Galβ1-4Glcβ (P_{k})	-0,149	Weak	0,023*
254	GlcNAcβ1-6(Galβ1-3) GalNAcα (core 2)	-0,219	Weak	< 0,001*
258	$(Man\alpha1)_2$-3,6Manβ-Gly	-0,137	Weak	0,035*
267	GlcNAcβ1-3Galβ1-3GlcNAcβ (GlcNAcβ3′Le^{C})	-0,202	Weak	0,002*
299	Neu5Acα2-3Galβ1- 3GlcNAcβ (3′SiaLe^{C})	-0,216	Weak	< 0,001*

* The difference is statistically significant ($p < 0,05$).

Table S5. The results of the correlation analysis between the levels of anti-glycan antibodies (IgM) and the age of healthy donors.

Glycan ID	Glycan (trivial name)	Significance of the correlation		
		ρ	Chaddock's Scale of Association	*p*
4	GalNAcα-O-Ser (Tn-Ser)	-0,359	Moderate	0,001*
5	GalNAcα (Tn)	-0,350	Moderate	0,002*
17	Manα-Gly	-0,421	Moderate	< 0,001*
18	Manβ-Gly	-0,433	Moderate	< 0,001*
36	Manβ-C3	-0,391	Moderate	< 0,001*
41	6-*O*-Su-GalNAcα	-0,390	Moderate	< 0,001*
52	Neu5Gcα	-0,384	Moderate	< 0,001*
75	Gala1-2Galb	-0,375	Moderate	< 0,001*
78	Galα1-3GalNAcα ($T_{\alpha\alpha}$)	-0,422	Moderate	< 0,001*
85	Galβ1-3GlcNAcβ (Le^{C})	-0,419	Moderate	< 0,001*
88	Galβ1-3GalNAcβ ($T_{\beta\beta}$)	-0,368	Moderate	0,001*
89	Galβ1-3GalNAcα (TF)	-0,356	Moderate	0,002*
113	GlcNAcβ1-3GalNAcα (core 3)	-0,439	Moderate	< 0,001*
114	GlcNAcβ1-3Manβ-Gly	-0,397	Moderate	< 0,001*
122	Manα1-6Manβ-Gly	-0,460	Moderate	< 0,001*
123	Manβ1-4GlcNAcβ-Gly	-0,416	Moderate	< 0,001*
172	Neu5Acα2-6GalNAcα (SiaTn)	-0,271	Weak	0,018*
174	Neu5Gcα2-6GalNAcα (Neu5GcTn)	-0,286	Weak	0,012*
222	Galα1-3Galβ1-4GlcNAcβ (Galilli)	-0,462	Moderate	< 0,001*
223	Galα1-4Galβ1-4Glcβ (P_k)	-0,293	Weak	0,010*
254	GlcNAcβ1-6(Galβ1-3)GalNAcα (core 2)	-0,404	Moderate	< 0,001*
258	$(Man\alpha1)_2$-3,6Manβ-Gly	-0,485	Moderate	< 0,001*
267	GlcNAcβ1-3Galβ1-3GlcNAcβ (GlcNAcβ3′Le^{C})	-0,416	Moderate	< 0,001*
299	Neu5Acα2-3Galβ1-3GlcNAcβ (3′SiaLe^{C})	-0,434	Moderate	< 0,001*

* The difference is statistically significant ($p < 0,05$).

Table S6. General comparison of the level of anti-glycan antibodies (IgG) in blood of gastric cancer patients and healthy donors.

Glycan ID	Glycan (trivial name)	Patients (n=235)			Donors (n=76)			*p*
		Me	Q_1	Q_3	Me	Q_1	Q_3	
41	6-*O*-Su-GalNAcα	554	227	1652	561	180	1369	0,361
85	Galβ1-3GlcNAcβ (Le^C)	298	92	858	549	202	1549	0,002*
88	Galβ1-3GalNAcβ ($T_{ββ}$)	251	82	1000	247	84	786	0,877
113	GlcNAcβ1-3GalNAcα (core 3)	364	92	934	244	100	986	0,815
222	Galα1-3Galβ1-4GlcNAcβ (Galilli)	582	167	1831	395	127	2376	0,658
223	Galα1-4Galβ1-4Glcβ (P_k)	328	95	893	334	148	805	0,940
267	GlcNAcβ1-3Galβ1-3GlcNAcβ (GlcNAcβ3′Le^C)	354	90	1163	696	229	2281	0,002*
299	Neu5Acα2-3Galβ1-3GlcNAcβ (3′SiaLe^C)	694	148	2509	1346	283	3576	0,004*

* The difference is statistically significant (p < 0,05).

Table S7. The level of anti-glycan antibodies (IgG) in blood of gastric cancer patients in different age groups.

Glycan ID	Glycan (trivial name)	Age group	Antibody levels			p
			Me	$Q_1 - Q_3$	n	
41	6-*O*-Su-GalNAcα	29-30	440	160 – 1240	4	0,553
		30-39	761	310 – 884	11	
		40-49	653	285 – 2557	20	
		50-59	545	208 – 1081	65	
		60-69	498	213 – 1392	83	
		70-80	929	306 – 1906	52	
85	Galβ1-3GlcNAcβ (Le^C)	29-30	247	186 – 1370	4	0,592
		30-39	220	183 – 334	11	
		40-49	376	105 – 746	20	
		50-59	411	132 – 1038	65	
		60-69	290	82 – 1262	83	
		70-80	188	90 – 599	52	
88	Galβ1-3GalNAcβ ($T_{\beta\beta}$)	29-30	145	119 – 205	4	0,269
		30-39	589	297 – 2244	11	
		40-49	264	92 – 984	20	
		50-59	254	91 – 1005	65	
		60-69	184	73 – 975	83	
		70-80	222	78– 793	52	
113	GlcNAcβ1-3GalNAcα (core 3)	29-30	1098	488 – 1716	4	0,730
		30-39	579	233 – 685	11	
		40-49	649	95 – 1271	20	
		50-59	384	136 – 969	65	
		60-69	298	87 – 744	83	
		70-80	324	95 – 806	52	
222	Galα1-3Galβ1-4GlcNAcβ (Galilli)	29-30	709	157 – 1388	4	0,407
		30-39	201	95 – 693	11	
		40-49	628	84 – 1611	20	
		50-59	508	170 – 1308	65	

		60-69	628	160 – 1820	83	
		70-80	821	233 – 2959	52	
223	Galα1-4Galβ1-4Glcβ (P_k)	29-30	413	287 – 585	4	0,509
		30-39	453	220,25 – 1207	11	
		40-49	448	152 – 1130	20	
		50-59	439	170 – 1000	65	
		60-69	323	82 – 773	83	
		70-80	228	82 – 734	52	
267	GlcNAcβ1-3Galβ1-3GlcNAcβ (GlcNAcβ3′Le^C)	29-30	439	341– 1880	4	0,379
		30-39	227	125 – 474	11	
		40-49	417	83 – 1121	20	
		50-59	502	119 – 1518	65	
		60-69	306	77 – 1253	83	
		70-80	276	80 – 1157	52	
299	Neu5Acα2-3Galβ1-3GlcNAcβ (3′SiaLe^C)	29-30	583	378 – 2939	4	0,693
		30-39	282	188 – 727	11	
		40-49	643	201 – 1802	20	
		50-59	962	184 – 2707	65	
		60-69	663	150 – 2618	83	
		70-80	541	118 – 2122	52	

Table S8. The level of anti-glycan antibodies (IgG) in blood of healthy donors in different age groups

Glycan ID	Glycan (trivial name)	Age group	Antibody levels			p
			Me	$Q_1 – Q_3$	n	
41	6-*O*-Su-GalNAcα	20-29	576	264– 903	25	0,914
		30-39	562	130 – 1675	32	
		40-49	634	187 – 1237	15	
		50-59	196	168 – 651	4	
85	Galβ1-3GlcNAcβ (Le^C)	20-29	901	337 – 2450	25	0,102
		30-39	549	233 – 1445	32	
		40-49	260	117 – 984	15	
		50-59	184	157 – 322	4	
88	Galβ1-3GalNAcβ ($T_{ββ}$)	20-29	250	142 – 466	25	0,506
		30-39	237	86 – 1184	32	
		40-49	93	77 – 438	15	
		50-59	1984	710 – 3717	4	
113	GlcNAcβ1-3GalNAcα (core 3)	20-29	192	154 – 500	25	0,388
		30-39	188	97 – 767	32	
		40-49	362	43 – 921	15	
		50-59	806	473 – 3269	4	
222	Galα1-3Galβ1-4GlcNAcβ (Galilli)	20-29	415	188 – 755	25	0,922
		30-39	433	176 – 3036	32	
		40-49	397	83 – 2972	15	
		50-59	232	1245 – 1969	4	
223	Galα1-4Galβ1-4Glcβ (P_k)	20-29	406	234 – 925	25	0,346
		30-39	336	134 – 892	32	
		40-49	219	81 – 589	15	
		50-59	197	136 – 280	4	
267	GlcNAcβ1-3Galβ1-3GlcNAcβ (GlcNAcβ3′Le^C)	20-29	1169	493 – 2921	25	0,095
		30-39	696	326 – 1334	32	
		40-49	298	127 – 1666	15	
		50-59	362	178 – 571	4	

299	Neu5Acα2-3Galβ1-3GlcNAcβ (3′SiaLe^{C})	20-29	2874	1178 – 5895	25	0,017*
		30-39	1638	882 – 2943	32	
		40-49	510	211 – 1925	15	
		50-59	249	162 – 505	4	

* The difference is statistically significant (p < 0,05).

Table S9. The results of the correlation analysis between the levels of anti-glycan antibodies (IgG) and the age of gastric cancer patients.

Glycan ID	Glycan (trivial name)	Significance of the correlation		
		ϱ	Chaddock's Scale of Association	*p*
41	6-*O*-Su-GalNAcα	0,075	Negligible	0,253
85	Galβ1-3GlcNAcβ (Le^C)	-0,065	Negligible	0,321
88	Galβ1-3GalNAcβ ($T_{ββ}$)	-0,049	Negligible	0,457
113	GlcNAcβ1-3GalNAcα (core 3)	-0,064	Negligible	0,328
222	Galα1-3Galβ1-4GlcNAcβ (Galilli)	0,115	Weak	0,077
223	Galα1-4Galβ1-4Glcβ (P_k)	-0,107	Weak	0,102
267	GlcNAcβ1-3Galβ1-3GlcNAcβ (GlcNAcβ3′Le^C)	-0,074	Negligible	0,257
299	Neu5Acα2-3Galβ1-3GlcNAcβ (3′SiaLe^C)	-0,028	Negligible	0,666

Table S10. The results of the correlation analysis between the levels of anti-glycan antibodies (IgG) and the age of healthy donors.

Glycan ID	Glycan (trivial name)	Significance of the correlation		
		ϱ	Chaddock's Scale of Association	p
41	6-*O*-Su-GalNAc*α*	0,016	Negligible	0,890
85	Galβ1-3GlcNAcβ (Le^{C})	-0,274	Weak	0,017*
88	Galβ1-3GalNAcβ ($T_{ββ}$)	-0,020	Negligible	0,864
113	GlcNAcβ1-3GalNAc*α* (core 3)	0,059	Negligible	0,611
222	Gal*α*1-3Galβ1-4GlcNAcβ (Galilli)	-0,039	Negligible	0,739
223	Gal*α*1-4Galβ1-4Glcβ (P_k)	-0,192	Weak	0,096
267	GlcNAcβ1-3Galβ1-3GlcNAcβ (GlcNAcβ3′Le^{C})	-0,284	Weak	0,013*
299	Neu5Ac*α*2-3Galβ1-3GlcNAcβ (3′SiaLe^{C})	-0,324	Moderate	0,004*

* The difference is statistically significant ($p < 0,05$).

Table S11. The comparison of the level of anti-glycan antibodies (IgM) in blood of gastric cancer patients and healthy donors in the 35-50 age group.

Glycan ID	Glycan (trivial name)	Patients (n=31)			Donors (n=30)			p
		Me	Q1	Q3	Me	Q1	Q3	
4	GalNAcα-O-Ser (Tn-Ser)	2444	824	4312	2840	1002	6339	0,863
5	GalNAcα (Tn)	3332	1300	6145	4621	2281	6728	0,371
17	Manα-Gly	811	400	1188	866	482	1373	0,751
18	Manβ-Gly	820	334	1021	730	422	1369	0,829
36	Manβ-C3	3285	1152	9455	5341	1530	9204	0,554
41	6-*O*-Su-GalNAcα	6549	4523	14358	6546	3448	11732	0,634
52	Neu5Gcα	886	263	1563	585	307	881	0,184
78	Galα1-3GalNAcα ($T_{\alpha\alpha}$)	3435	1355	8088	2873	1522	4822	0,545
85	Galβ1-3GlcNAcβ (Le^C)	4487	1202	13397	7598	2590	13236	0,545
88	Galβ1-3GalNAcβ ($T_{\beta\beta}$)	4389	2501	11522	6623	4499	11352	0,105
89	Galβ1-3GalNAcα (TF)	2331	1153	3601	2534	950	8031	0,498
113	GlcNAcβ1-3GalNAcα (core 3)	5420	3495	10131	6213	2729	15486	0,644
114	GlcNAcβ1-3Manβ-Gly	1092	424	3796	1433	667	4124	0,498
122	Manα1-6Manβ-Gly	678	422	1297	627	330	878	0,282
123	Manβ1-4GlcNAcβ-Gly	5568	2324	11961	5139	2153	9824	0,574
172	Neu5Acα2-6GalNAcα (SiaTn)	3081	977	5202	3046	1380	4522	0,863
174	Neu5Gcα2-6GalNAcα (Neu5GcTn)	3418	1181	6167	3455	1762	5300	0,697
222	Galα1-3Galβ1-4GlcNAcβ (Galilli)	3514	1939	8144	3969	2319	6877	0,697
223	Galα1-4Galβ1-4Glcβ (P_k)	6140	3158	9283	6646	2147	13344	0,614
254	GlcNAcβ1-6(Galβ1-3)GalNAcα (core 2)	2065	1273	5117	2673	801	6116	0,908

258	(Manα1)$_2$-3,6Manβ-Gly	851	379	1391	657	398	1049	0,564
267	GlcNAcβ1-3Galβ1-3GlcNAcβ (GlcNAcβ3′Le^C)	4480	1482	12624	7320	2665	12795	0,379
299	Neu5Acα2-3Galβ1-3GlcNAcβ (3′SiaLe^C)	4136	1648	20726	7103	2668	13745	0,593

Table S12. The comparison of the level of anti-glycan antibodies (IgG) in blood of gastric cancer patients and healthy donors in the 35-50 age group.

Glycan ID	Glycan (trivial name)	Patients (n=31)			Donors (n=30)			*p*
		Me	Q1	Q3	Me	Q1	Q3	
41	6-*O*-Su-GalNAcα	677	275	2389	554	109	1527	0,231
85	Galβ1-3GlcNAcβ (Le^{C})	290	110	707	434	187	1488	0,123
88	Galβ1-3GalNAcβ ($T_{ββ}$)	292	96	1134	214	77	845	0,162
113	GlcNAcβ1-3GalNAcα (core 3)	579	98	873	244	87	632	0,149
222	Galα1-3Galβ1-4GlcNAcβ (Galilli)	434	86	1450	389	84,25	2599	0,902
223	Galα1-4Galβ1-4Glcβ (P_k)	453	183	1347	260	98	698	0,145
267	GlcNAcβ1-3Galβ1-3GlcNAcβ (GlcNAcβ3′Le^{C})	291	85	729	685	198	1983	0,075
299	Neu5Acα2-3Galβ1-3GlcNAcβ (3′SiaLe^{C})	448	1778	1523	1278	218	3643	0,103
